# Survival of *Salmonella* spp., *Escherichia coli* O157:H7, and *Listeria monocytogenes* in Ready-to-Eat “Guacamole”: Role of Added Antimicrobials

**DOI:** 10.3390/foods13142246

**Published:** 2024-07-17

**Authors:** Rameez Al Daour, Tareq M. Osaili, Lucy Semerjian, Dinesh Kumar Dhanasekaran, Leila Cheikh Ismail, Ioannis N. Savvaidis

**Affiliations:** 1 Department of Environmental Health Sciences, College of Health Sciences, University of Sharjah, Sharjah 27272, United Arab Emirates; rameezdaour@hotmail.com (R.A.D.); lsemerjian@sharjah.ac.ae (L.S.); 2 Research Institute of Medical and Health Sciences (RIMHS), University of Sharjah, Sharjah 27272, United Arab Emirates; tosaili@sharjah.ac.ae (T.M.O.); ddhanasekaran@sharjah.ac.ae (D.K.D.); lcheikhismail@sharjah.ac.ae (L.C.I.); 3 Department of Clinical Nutrition and Dietetics, College of Health Sciences, University of Sharjah, Sharjah 27272, United Arab Emirates; 4 Department of Nutrition and Food Technology, Faculty of Agriculture, Jordan University of Science and Technology, Irbid 22110, Jordan; 5 Research Institute of Science and Engineering, University of Sharjah, Sharjah 27272, United Arab Emirates; 6 Nuffield Department of Women’s & Reproductive Health, University of Oxford, Oxford OX1 2JD, UK; 7 Laboratory of Food Chemistry and Food Microbiology, Department of Chemistry, University of Ioannina, 45110 Ioannina, Greece

**Keywords:** guacamole, chitosan, mastic oil, citric acid, foodborne pathogens, natural preservatives, temperature abuse

## Abstract

Ensuring the microbiological safety of food products is majorly important to regulatory agencies, producers, and consumers. This study aimed to examine the effects of three different antimicrobial agents, including chitosan (CH), mastic oil (M), and citric acid (CA), individually or as a combination, against *Salmonella* spp., *Escherichia coli* O157:H7, and *Listeria monocytogenes* (artificially inoculated) in Guacamole, a ready-to-eat (RTE) avocado-based salad. The Guacamole samples included untreated samples, designated as CNL, and samples treated as follows: CA 0.15% and CA 0.30% with citric acid added at 0.15% and 0.30% *v*/*w*; CH 0.5% and CH 1% with chitosan at 0.5 and 1% *v*/*w*; M 0.2% and M 0.4% with mastic essential oil (EO) at 0.2% and 0.4% *v*/*w*; CACH with CA 0.30% and CH 1% *v*/*w*; CAM with CA 0.30% and M 0.4% *v*/*w*; CHM with CH 1% and M 0.4% *v*/*w*; and CACHM with CA 0.30%, CH 1%, and M 0.4% *v*/*w*. Microbiological evaluation, monitoring of the pH values, and proximate analyses (moisture, fat, protein, ash, and water activity) were performed at different time intervals (days 0, 1, 3, 5, and 7) at two storage temperatures (4 and 10 °C). Antimicrobial treatments, particularly CH 1% and CACHM, effectively (*p* < 0.05) reduced *Salmonella* spp. and *E. coli* O157:H7 populations at 4 °C, while CACHM showed the most efficacy against *L. monocytogenes*. However, at 10 °C, antimicrobials had limited impact, and the bacterial counts exhibited an increasing trend during storage. The pH values in the avocado-based salad samples showed, in general, higher decreases at 10 compared to 4 °C, with the CHM combination showing the highest antimicrobial effect.

## 1. Introduction

Foodborne pathogens, especially bacterial species *Campylobacter jejuni*, *Clostridium perfringens, Escherichia coli*, *Listeria monocytogenes*, *Salmonella* spp., and *Staphylococcus aureus,* may potentially be present in RTE salads and, therefore, in certain cases (e.g., immunocompromised, elderly consumers), lead to foodborne illnesses [1]. Over the past decade, salad dips and appetizers have gained popularity and are often consumed without any further processing (thermal treatment). However, improper food handling, inefficient refrigeration, etc., can pose significant safety concerns for these products. Therefore, preventing microbiological contamination from foodborne pathogens entering at any stage of the food-processing chain is crucial.

Avocado (Persea Americana) belongs to the Lauraceae family, with “Hass avocados” being the most widely consumed variety worldwide. This variety is notably known for its unique fat (fatty acids) and vitamin E contents, both considered essential nutrients, offering several health benefits [2].“Guacamole” and salsa are examples of RTE salads or side dishes, widely consumed in the Americas (especially, North and Central), Europe, and other areas, including the Middle East. RTE avocado-based salads, served, in most cases, as side dishes or appetizers (“Guacamole”, prepared with fresh avocado, together with other added ingredients), can potentially be contaminated by low-quality raw materials, food handlers, or even utensils during their preparation. Therefore, avocado-based salad products’ safety depends on applying good manufacturing practices and effective temperature control in order to ensure, ideally, the absence of foodborne pathogenic bacteria [3].

There is cause for concern due to the high morbidity associated with the *Salmonella* spp. and fatality due to *E. coli* O157:H7 and *L. monocytogenes* [4]. Contamination of avocado-based salad formulations, like any other perishable RTE product, can take place at any point in the chain of production. In Mexico, previous reports have indicated that *Salmonella* spp. and *L. monocytogenes* were isolated in 3% (4/144) and 0.7% (1/144), respectively, from farm-collected Hass avocados [5]. *Salmonella* spp. were also found in environmental samples, with a frequency of 4% (27/720) in soil, 9% (16/180) in water, and 3% (5/163) in workers’ shoes, door handles, and harvesting utensils [5]. In addition, the outer skin of whole avocados in Mexico (*n* = 225), sold at local retail markets, showed a frequency of 4 and 8% for *Salmonella* spp. and *L. monocytogenes*, respectively [6]. According to a US study, *Salmonella* spp. and *L. monocytogenes* prevalence in whole fresh avocados, before reaching retail stores, was 0.7% (*n* = 1615) and 18% (*n* = 361), respectively [1]. As demonstrated in these studies, *Salmonella* spp. and *L. monocytogenes* can be found on whole fresh avocado’s skin, and there was a risk identified of those pathogens transferring into the fruit pulp, via punctures, wounds, or cuts [7]. Extending raw avocados’ or its product formulations’ shelf-life and additionally assuring their safety both present challenges to the food-processing industries, in view of consumers’ current demands for foods without the use of chemical fungicides and preservatives. Public authorities have also encouraged the food industry to reduce the usage of chemical preservatives and seek natural methods of food preservation. This demand, coupled with a huge social request for “clean label” food products which are more “natural,” less heavily processed, and safe [8], initiated research into the concept of “Food Natural Antimicrobials” among researchers over the last 15 years. Food natural antimicrobials derived from plants or other sources are already seeing applications, and foods with added antimicrobials may prove themselves to be nutritious, safe, and with less toxic ingredients, features which are all desired by consumers [9].

Natural antimicrobials have been investigated in a range of plant-based foods to evaluate their use in assuring food quality and safety, including in eggplant dips [10], salad dips [11], hummus [12], tahini [13], and animal-sourced food products such as chicken [14]. However, a limited number of studies have investigated the survival of pathogens of concern (e.g., *Salmonella* spp., *E. coli* O157:H7, *L. monocytogenes*) potentially found in avocado-based salad products. Moreover, the effects of adding natural antimicrobials (e.g., chitosan, citric acid, mastic oil) on foodborne pathogens (artificially inoculated) in avocado salad dip have been limited or not examined. Therefore, the present study was undertaken to evaluate the following: (a) the survival of selected foodborne bacterial species (*Salmonella* spp., *Listeria monocytogenes,* and *E. coli* O157: H7), added in a Guacamole salad (in the absence of antimicrobials); (b) the effects of the addition of different antimicrobials (citric acid, mastic oil, chitosan) on the survival of *Salmonella* spp., *L. monocytogenes,* and *E. coli* O157: H7 in the salad samples; and (c) the effects of temperature conditions of 4 and 10 °C (refrigerated and mild abuse), over a storage period of 7 days.

## 2. Materials and Methods

### 2.1. Preparation of the Guacamole RTE Salad

Fresh, Mexican (“Huss” variety) avocadoes, cilantro, tomatoes, onions, jalapenos, and salt were all purchased from a local supermarket (Sharjah, UAE). Avocados were stored at room temperature (24 °C) for 2 days to ripen, while the remaining ingredients, including tomatoes, cilantro, and lemons, were refrigerated at 4 °C. The onions and salt were stored at room temperature. The avocados were soaked and rubbed in sterile water, washed in a fruits and vegetables wash, peeled under aseptic conditions, and subsequently mashed well with the remaining ingredients until the desired dip consistency was achieved (a paste-like mixture). Guacamole was prepared by mixing the ingredients with a sterile spoon. The ingredients were as follows: mashed avocado pulp (71% *w*/*w*), diced tomato (10% *w*/*w*), diced onion (8% *w*/*w*), lemon juice (5% *v*/*w*), a diced jalapeno pepper (3.5% *w*/*w*), chopped cilantro (1.5% *w*/*w*), and salt (1% *w*/*w*) [15].

### 2.2. Media, Chemicals, and Culture Preparation

All media and supplements used in the microbiological analysis were acquired from Himedia (Himedia, Mumbai, India). Chemical materials (chitosan and citric acid—monohydrate form) were purchased from Sigma-Aldrich Chemie, Schenelldorf, GmbH, Germany. The chitosan solution was prepared from low-molecular-weight chitosan (50–160 kDa) with a deacetylation degree of 75−85% (Sigma-Adrich Chemie). Deionized water was used to make chitosan final concentrations of 0.5 and 1% (*w*/*w*). To the chitosan solutions, an acetic acid solution (0.33% *v*/*v*) was also added. Citrate solutions were made using deionized water to final concentrations of 0.15 and 0.30% *w*/*v*. Mastic EO in pure form (organic) was purchased from Masticha shop (Chios, Greece). A microbiological analysis was conducted, analyzing the effect of selected antimicrobials at different concentrations on the pathogens *Salmonella* spp., *E. coli* O157:H7, and *L. monocytogenes*. Strains of *Salmonella enterica* (S. Copenhagen PT 99 and S. Typhimurium 02-8423), *Escherichia coli* O157:H7 (O157:H7 1934 and O157:H7 161-84), and *Listeria monocytogenes* (L. 7644 and L.M. GLM 5) were obtained from Agriculture and Agri-Food Canada, Food Research Institute, Ottawa, and Health Canada, Microbial Hazards Laboratory, Ottawa, Canada. Strains were grown individually in sterile Tryptone Soy broth (TSB, 10 mL) with added 0.6% (*w*/*v*) yeast extract (10 mL). All tubes were incubated at 37 °C for 24 h. Each culture was centrifuged (Herolab, UniCen M, Wiesloch, GmbH, Germany) at 3000× *g* for 20 min at 21 °C, and the pellet was resuspended in 1 mL sterile peptone water.

### 2.3. Culture Inoculation and Addition of Antimicrobials to the Guacamole RTE Salad

**Figure 1:**
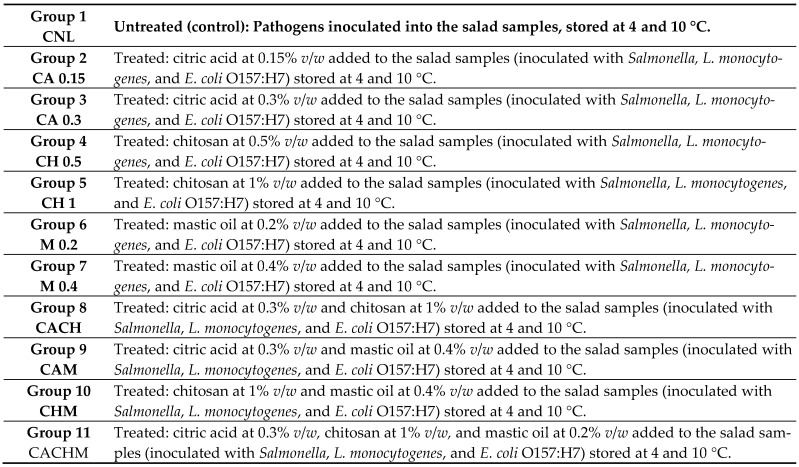
Antimicrobial treatments of the RTE Guacamole salad.

Before conducting the experiments, the primary ingredients (tomato, onion, lemon, jalapeno pepper, cilantro, and salt) used for the Guacamole salad’s preparation were tested, according to standard ISO methods, for the presence of *Salmonella* spp, *Escherichia coli* O157:H7, and *Listeria* spp. The ingredients were found to be free from these bacteria, which were subsequently inoculated into the Guacamole salad [13,14]. Three different cocktail mixtures (*Salmonella* spp., *E. coli* O157:H7, and *L. monocytogenes*) were prepared by mixing each strain (1 mL). Each bacterial mixture (0.1 mL) was individually added to 10 g of Guacamole, thoroughly mixed using a sterile spoon, resulting in final cell populations of approximately 7 log CFU/mL in the salad. The samples were subsequently stored in sterile air-tight containers (50 mL capacity) in a refrigerator for 20 min to allow the attachment of the cells to the salad. Antimicrobials, either as a single treatment or as a combination, at different concentrations, were added to the samples, as shown in Figure 1. Two lots—A and B, untreated and treated, respectively—were used: Lot A (untreated) comprised control Guacamole samples, inoculated with the pathogen, designated as CNL. Lot B (treated) consisted of Guacamole samples, inoculated with each pathogen culture and added (single or combination) antimicrobials, designated as follows: CA 0.15%, with citric acid at 0.15% *v*/*w*; CA 0.30%, with citric acid at 0.30% *v*/*w*; CH 0.5%, with chitosan at 0.50% *v*/*w*; CH 1%, with chitosan at 1% *v*/*w*; M 0.2%, with mastic oil at 0.2% *v*/*w*; M 0.4%, with mastic oil at 0.4% *v*/*w*; CACH, with citric acid at 0.30% *v*/*w* and chitosan at 1% *v*/*w*; CAM, with citric acid at 0.30% *v*/*w* and mastic oil at 0.4% *v*/*w*; CHM, with chitosan at 1% *v*/*w* and mastic oil at 0.4%; and CACHM, with citric acid at 0.30% *v*/*w*, chitosan at 1% *v*/*w,* and mastic oil at 0.4% *v*/*w*. All the inoculated Guacamole samples, including untreated (absence of antimicrobials) and treated (presence of antimicrobials) ones, were stored at two selected temperature conditions (4 °C, refrigerated; and 10 °C, mild abuse) over a 7-day period. Sampling was conducted at predetermined time intervals on days 0, 1, 3, 5, and 7. In total, this study involved three independent (separately conducted) replicate experiments. In each experiment, approximately 1350 g of Guacamole salad was used.

### 2.4. Microbiological and Chemical Analyses

Guacamole samples (10 g) were placed in sterile stomacher bags (Seward Co., Worthing, UK) with 90 mL of sterile peptone water, and the mixture was homogenized for 2 min using a Stomacher (Easy Mix, AES Laboratories, Bruz, France). The enumeration of *Salmonella* spp., *E. coli* O157:H7, and *L. monocytogenes* was determined by spread plating 0.1 mL of diluted samples onto selective media. Xylose Lysine Deoxycholate (XLD) agar was used for the enumeration of *Salmonella* spp.; Sorbitol MacConkey (SMAC) agar was used for the enumeration of *E. coli* O157:H7; and Listeria Selective Agar (Oxford Formulation) (HiMedia Laboratories Private Limited, Maharashtra, India) was used for the enumeration of *L. monocytogenes*. The plates were incubated under aerobic conditions at 37 °C after inoculation, and the cell colonies were counted after 48 h. Microbiological data are reported as log CFU/g. In addition, the Guacamole samples were also subjected to lipid oxidation and proximate analysis (moisture, ash, crude protein, and crude fat content) with the methods previously described by Al-Nabulsi et al. [13] and Osaili et al. [14]. Water activity (aw) was initially assessed with a aw meter at the onset of the experiment (Hygrolab, Rotronic Instrument Corp., Huntington, NY, USA). The pH of the samples was determined using a pH meter (Cyberscan 500; Eutech Instruments, Singapore). Each separate replication (3 experiments) used 1350 g Guacamole

### 2.5. Statistical Analysis

Statistical tests were conducted to determine the efficacy of antimicrobial treatments, storage time, and their interacting effects on the survival of *Salmonella* spp., *E. coli* O157:H7, and *L. monocytogenes* in the Guacamole salad. A two-way analysis of variance (ANOVA) and a post hoc analysis using Tukey’s HSD were performed for the microbiological and pH analyses using the IBM SPSS Statistics software program (version 26, Chicago, IL, USA). Furthermore, *t*-tests were conducted to determine whether there were statistically significant differences between the storage temperatures, and a *p*-value of less than or equal to 0.05 (*p* < 0.05) was used.

## 3. Results

Proximate analyses of the avocado product used in our study gave moisture, fat, protein, ash, and water activity values of 79.5%, 1.3%, 8%, 2%, and 0.86, respectively. The presence of *Salmonella* spp., *E. coli* O157:H7, and *L. monocytogenes* in the Guacamole salad, prior to inoculation, was evaluated via the direct plating technique, and none of the aforementioned pathogens were detected.

The antimicrobials CH 1%, CACH, and CAM reduced *Salmonella* growth at 4 °C but increased it at 10 °C. Notably, CACHM demonstrated low growth between the temperatures on day 1, indicating different antimicrobial efficacy under various temperature conditions. Overall, CH 1%, CACH, and CACHM displayed the most effective antimicrobial action against *Salmonella* spp. at 4 °C, while growth increased at 10 °C across all intervals. With regard to *E. coli* O157:H7, antimicrobial treatments at 4 °C resulted in significant reductions in *E. coli* O157:H7 counts, particularly with CH1% and CACHM, exhibiting an average decrease of 0.45 log CFU/g by day 7. However, at 10 °C, the *E. coli* O157:H7 counts displayed an increasing trend, irrespective of antimicrobial presence. The addition of antimicrobial treatments at 4 °C showed slight reductions in *L. monocytogenes* counts, with CH1% and CACHM demonstrating the most efficacy. Despite the increase in CNL at both temperatures, at 10 °C, the antimicrobials had a minimal effect on the *L. monocytogenes* counts, with slight reductions observed for some treatments on day 7, but not sustained throughout storage. In summary, Table 1, Table 2 and Table 3 show that there are interaction and temperature effects (Table 2, *E. coli* O157:H7 only), but there is only a temperature effect between the storage temperatures (4 and 10 °C) for *Salmonella* spp and *L. monocytogenes* (Table 1 and Table 3, respectively).

There was a significant decline in pH from day 0 to day 7 at 10 °C (Table 4). However, at 4 °C, slight pH decreases were found. The control (CNL) pH values (7.2–7.6) of the Guacamole samples remained practically unchanged at 4 °C. Furthermore, the nutrient competition between the pathogens and natural microflora pH could have contributed significantly to the reduction in pH over the day intervals that was noted at 10 °C.

## 4. Discussion

Avocadoes are considered fruits, and their principal ingredient, fat, provides several health benefits to humans. “Hass” avocados are probably the most popular variety, consumed all over the world. Data from our study show that, in the absence or presence of antimicrobials, the experimentally inoculated pathogens, during the entire storage period (7-day), did not reach high numbers (to a greater extent at 4 °C), with their final populations barely in the range of 5.5–4.6 (4 °C) or 7.0–6.0 (10 °C) logs in the Guacamole samples.

Growth of the inoculated *Salmonella* spp., *E. coli* O157:H7, and. *L. monocytogenes* in the Guacamole was greatly limited (to a greater extent, as expected, at 4 °C as opposed to 10 °C). Moreover, it is likely that certain ingredients used to prepare the Guacamole salad (tomatoes, cilantro, and onions) may possess an antibacterial function. The tomato, cilantro, jalapeno peppers, and onions (being rich in flavanols, e.g., quercetin) added to the salad could present an antimicrobial consortium that prevented the bacterial species in our study from reaching high numbers. Such a statement, however, can only be speculated upon (as no investigation was conducted in our study). Indeed, in a relevant study [16], it was also speculated that the addition of fresh tomatoes, garlic, and lemon juice in a similar Guacamole formulation inhibited *Salmonella* spp. from growing during storage at room temperature. Differences in the acidity (pH) values, storage period, and temperatures as well as in the ingredients and inoculated strains could probably account for variations in the behavior of these species in avocado-based RTE salads (e.g., salsa, Guacamole).

*L. monocytogenes* presented a higher growth at 10 °C compared to 4 °C in the Guacamole on the final day (day 7) (a 1.5 logs difference recorded). Bacterial counts between 5 and 7 logs are considered a food safety risk, and the unlikely event of such a high number being initially present or caused by post-processing contamination conditions would result in a high-risk and unsafe product, especially for certain vulnerable populations [17]. It must be noted that the initial survival of pathogens or their subsequent growth depends on the ability of the foodborne pathogens to adapt to the food matrix’s nutrients and on the existing, extrinsic parameters of its immediate environment. Recent findings have shown that the *Salmonella* spp. is able to penetrate through the intact avocado peel, not only reaching various sections of the fruit but also being capable of growing at various points during its shelf-life [10]. Additionally, it has been previously shown that pathogens, including *Salmonella* spp. and *L. monocytogenes*, can enter the avocado pulp and are able to survive and even proliferate in RTE salads (hummus, tahini, baba ghanoush, Guacamole, and pesto), posing a considerable risk to certain consumers [1,18,19,20,21].

In the case of *L. monocytogenes*, which have the ability to resist both low refrigeration conditions and grow at mesophilic temperatures [22], the likelihood of their survival and growth in RTE salads, including Guacamole, cannot be excluded. Iturriaga et al. [23] found that *L. monocytogenes* inoculated in avocado pulp and processed Guacamole stored at 22 °C reached ca. 9 and 8 log CFU/g, respectively, after 48 h. These authors suggested that high-pressure processing could be useful as a technology for the reduction in *L. monocytogenes* in certain RTE salad dips, including hummus, Guacamole, and baba ghanoush; however, under the conditions tested, HPP was ineffective in pesto and tahini [23].

Our results show that, at 4 °C, the growth of *L. monocytogenes* was not limited in the untreated (CNL) samples, whereas *Salmonella* spp. and *E. coli* O157:H7 growth was significantly inhibited. Mild temperature abuse (10 °C) allowed these pathogens to reach high numbers in a Guacamole product [24] after a 12 h storage period at room temperature. On the contrary, Neetoo et al. observed that *Salmonella* spp. could not grow in Guacamole (unacidified and acidified) [16]. These authors noted that the fate of *Salmonella* spp. during storage is dependent on the strains employed in its inoculation as well as the type of ingredients used in the salsa or Guacamole preparations [16]. Variations in the ingredients used for the Guacamole formulations could account for the differences recorded in our data compared to other studies.

It is well established, based on numerous studies over the last decade, that the addition of mostly plant-derived antimicrobials (EOs) or other antimicrobials derived from bacteria (nisin) and crustaceans (chitosan) may exert an antimicrobial action and, when applied alongside novel food-processing technologies (high-pressure processing, electrolyzed oxidizing water, aqueous chlorine dioxide, etc.), present a more effective means of inhibiting, limiting, or even eliminating foodborne pathogens such as *Salmonella* spp., *E. coli* O157:H7, and *L. monocytogenes* in various types of foods or food products [15]. Similarly, in a recent study, *Salmonella* spp., *E. coli* O157:H7, and *L. monocytogenes* in hummus dip [7,25] were inhibited by chitosan, with an increase in the shelf-life. Regarding the selected natural antimicrobials added, either by themselves or in combination, to the Guacamole, the present results show that no significant reductions were recorded in *Salmonella* spp. or *E. coli* O157:H7 numbers (day 7), with the exception of chitosan (1%) and the CACHM combination, only at 4 °C.

Citric acid or acetic acids (predominant components in lemon juice or vinegar, respectively) are usually added to Guacamole or salsa to impart flavor and maintain a desirable pH range (usually pH 3.8–4.6). Citric acid, added at levels of 0.15 and 0.3% *v*/*w* to the Guacamole, did not seem to affect the initial acidity (pH values 6.3–7.2) of the product formulation, which probably accounts for its ineffectiveness in causing reductions in the cell numbers of the inoculated bacterial species tested at both 4 and 10 °C. It must be stated that, in general, the effectiveness of citric or organic acids may depend on a product’s ingredients as well as its intrinsic properties (pH, water activity, fat content, etc.). In other studies, Osaili et al. reported a decrease in *Salmonella* spp. counts in eggplant dip samples with citric acid, compared to the control samples [25], whereas Al-Rousan et al. showed that acetic acid (0.4%) was more inhibitory toward S. Typhimurium and *E. coli* O157:H7 than citric acid in tabbouleh salad stored at 21 °C [26].

The antimicrobial activity of the EO of mastic gum (*Pistacia lentiscus* var. *chia*) on Gram-positive and Gram-negative bacteria in broth and a model food system has been previously demonstrated [27]. In our study, mastic oil’s action was, in general, limited when applied as a single treatment (M) against all the species tested, resulting in minor decreases of 0.2–0.3 logs (*Salmonella* spp, *E. coli* O157:H7, 4 °C). Out of the combined antimicrobial treatments tested, it appears that only mastic oil with chitosan (CHM) was able to achieve reductions (<1 log CFU), irrespective of the storage temperature.

Chitosan, known for exerting an effective antimicrobial action, together with novel food-processing technologies, EOs, packaging, etc., may extend the shelf-life or inhibit, delay, and/or eliminate foodborne pathogenic bacteria such as *Salmonella* spp., *E. coli* O157:H7, *L. monocytogenes*, etc. [28]. It has previously been reported that fat has no effect on chitosan’s antimicrobial activity, which could be explained by the fact that chitosan is positioned on the outside of the emulsion drops due to the interaction between the positively charged chitosan and the negatively charged free fatty acids [29]. According to Jumaa et al., chitosan has a higher antimicrobial activity in oily foods [30]. The use of chitosan, either by itself or as a combined antimicrobial treatment, in avocado product formulations (salsa, Guacamole) has not yet been reported in the available literature. The present data (based on the CH, CHM, and CACHM treatments) show that chitosan, either added as a single treatment or as part of a combined intervention, could potentially be considered an effective natural antimicrobial agent against the growth of *Salmonella* spp. and/or *E. coli* O157:H7 in Guacamole RTE salads. In other studies, a chitosan coating on white, brined cheese reduced *E. coli* O157:H7 by 2.8 and 2.1 log CFU/g at 4 and 10 °C [31].

Except for the control (CNL) (4, 10 °C), the pH values followed a declining trend for the treated samples. In our study, the pH of Guacamole was left unacidified (pH values: 7.2, at 4 °C, and 6.3, at 10 °C, on the initial day; CNL samples) in contrast to the pH of salsa and Guacamole side dishes (adjusted to pH 4–3.8 and 4.6–4.3, with lemon juice or vinegar) [16]. Moreover, Bell et al. indicated that the minimum growth pH for the *Salmonella* spp. is 3.8, which may partly explain the ability of the *Salmonella* spp. strains in our study to grow in the Guacamole samples [32].

It should be noted that the degree of bacterial inactivation will vary depending on the strain(s) employed, the type of acid present in the avocado side dish, and other prevailing conditions (intrinsic/extrinsic). As a result, the fate of foodborne pathogens such as those in our investigation might be said to rely on the strains employed in the inoculation, the makeup of the ingredients in the avocado formulation, and the antimicrobials (if utilized). In view of the potential of chitosan as a promising antimicrobial agent [33], it is a challenge for the food-processing industry to test formulations of chitosan, either by itself or in combination with other antimicrobials (e.g., EOs), to extend the shelf-life of products and also provide increased safety against foodborne pathogens potentially surviving or growing in RTE salads, including in Guacamole-based products.

## 5. Conclusions

Our results show that *Salmonella* spp., *E. coli* O157:H7, and *L. monocytogenes*, either in the absence or presence of selected antimicrobials, were able to survive in an RTE Guacamole salad over a storage period of 7 days at 4 and, showing an increasing trend, 10 °C. It could be speculated that the avocado’s lipid content in the pulp (15–20%), the added ingredients, and their initial microbiota might have hindered the evolution of these species, preventing them from reaching high numbers. It is, thus, important to assess the potential for *Salmonella* spp, *E. coli* O157:H7, and *L. monocytogenes* to survive in avocado-based salad formulations (dip, salsa, etc.) and understand the factors that support their survival in such RTE products. Further research is needed to establish threshold natural antimicrobial limit values in order to achieve desirable and acceptable (organoleptically) products that meet quality and safety standards. Our study, for the first time, provides information on the use of natural antimicrobials against the survival/growth of *Salmonella* spp, *E. coli* O157:H7, and *L. monocytogenes* in an avocado-based RTE salad.

## Figures and Tables

**Table 1:** Effect of antimicrobial agents on the growth of *Salmonella* spp. (log CFU/g) in Guacamole during storage at 4 °C and 10 °C, for 7 days.

	Days	CNL	CA 0.15	CA 0.3	M 0.2	M 0.4	CH 0.5	CH 1	CACH	CAM	CHM	CACHM	*p*-Value
Temp 4 °C	Day-0	5.29 ± 0.12	5.16 ± 0.31	5.16 ± 0.31	5.16 ± 0.31	5.16 ± 0.31	5.16 ± 0.31	5.16 ± 0.31	5.16 ± 0.31	5.16 ± 0.31	5.16 ± 0.31	5.16 ± 0.31	NS
	Day-1	5.01 ± 0.45	4.68 ± 0.39	4.71 ± 0.24	4.70 ± 0.40	4.55 ± 0.34	4.67 ± 0.04	4.75 ± 0.28	4.67 ± 0.02	4.65 ± 0.14	4.81 ± 0.21	4.57 ^A^ ± 0.39	
	Day-3	5.41 ± 0.29	5.05 ^A^ ± 0.20	4.9 ± 0.12	5.05 ± 0.24	4.90 ± 0.12	5.02 ^A^ ± 0.23	4.83 ± 0.10	4.79 ± 0.13	4.78 ± 0.15	4.76 ± 0.17	4.51 ± 0.09	
	Day-5	5.35 ± 0.43	5.19 ± 0.59	5.17 ± 0.67	5.10 ± 0.58	5.02 ± 0.68	5.10 ± 0.59	5.06 ± 0.64	5.07 ± 0.58	5.03 ± 0.62	5.12 ± 0.54	4.94 ± 0.72	
	Day-7	5.43 ^A^ ± 0.38	4.98 ± 0.33	4.87 ^A^ ± 0.32	4.98 ^A^ ± 0.33	4.82 ^A^ ± 0.37	4.96 ^A^ ± 0.31	4.70 ^A^ ± 0.26	4.73 ^A^ ± 0.15	4.86 ^A^ ± 0.28	4.80 ^A^ ± 0.26	4.60 ^A^ ± 0.30	
Temp 10 °C	Day-0	5.31 ± 0.44	5.14 ± 0.24	5.14 ± 0.24	5.14 ± 0.24	5.14 ± 0.24	5.14 ± 0.24	5.14 ± 0.24	5.14 ± 0.24	5.14 ± 0.24	5.14 ± 0.24	5.14 ± 0.24	*p* < 0.05 NS
	Day-1	6.2 ± 0.93	5.92 ± 0.69	5.91 ± 0.66	6.15 ± 0.89	5.55 ± 0.58	6.04 ± 0.80	5.60 ± 0.86	5.75 ± 0.72	6.20 ± 0.93	5.71 ± 0.26	5.71 ^B^ ± 0.49	
	Day-3	6.4 ± 0.53	6.20 ^B^ ± 0.52	6.27 ± 0.60	6.20 ± 0.68	6.03 ± 0.58	6.29 ^B^ ± 0.50	6.13 ± 0.58	5.99 ± 0.63	6.07 ± 0.66	6.00 ± 0.69	5.95 ± 0.65	
	Day-5	6.28 ± 1.16	5.90 ± 0.70	5.86 ± 0.64	5.50 ± 0.75	5.43 ± 0.95	5.60 ± 0.81	5.50 ± 0.75	5.73 ± 0.57	5.80 ± 0.60	5.61 ± 0.41	5.62 ± 0.39	
	Day-7	7.06 ^B^ ± 0.56	6.26 ± 0.75	6.50 ^B^ ± 0.17	6.50 ^B^ ± 0.20	6.33 ^B^ ± 0.47	6.56 ^B^ ± 0.25	6.26 ^B^ ± 0.40	6.20 ^B^ ± 0.62	6.30 ^B^ ± 0.43	6.10 ^B^ ± 0.40	6.06 ^B^ ± 0.40	
Temp 4 °C Vs. Temp 10 °C	Day-0	NS	NS	NS	NS	NS	NS	NS	NS	NS	NS	NS	
	Day-1	NS	NS	NS	NS	NS	NS	NS	NS	NS	NS	0.03	
	Day-3	NS	0.05	NS	NS	NS	0.03	NS	NS	NS	NS	NS	
	Day-5	NS	NS	NS	NS	NS	NS	NS	NS	NS	NS	NS	
	Day-7	0.02	NS	0.00	0.01	0.01	0.00	0.00	0.05	0.01	0.01	0.01	

^A,B^ Different letters in each column indicate a significant difference (*p* < 0.05) between the means on the same day of storage at 4 and 10 °C. CNL: control; CA 0.15: citric acid 0.15%; CA 0.3: citric acid 0.3%; M 0.2: mastic oil 0.2%; M 0.4: mastic oil 0.4%; CH 0.5: chitosan 0.5%; CH 1: chitosan 1%; CACH: citric acid 0.3% + chitosan 1%; CAM: citric acid 0.3% + mastic oil 0.4%; CHM: chitosan 1% + mastic oil 0.4%; CACHM: citric acid 0.3% + chitosan 1% + mastic oil 0.4%. Significantly different at (*p* < 0.05); NS: non-significant.

**Table 2:** Effect of antimicrobial agents on the growth of *E. coli* O157:H7 (log CFU/g) in Guacamole during storage at 4 °C and 10 °C, for 7 days.

	Days	CNL	CA 0.15	CA 0.3	M 0.2	M 0.4	CH 0.5	CH 1	CACH	CAM	CHM	CACHM	*p*-Value
Temp 4 °C	Day-0	5.14 ^abcd^ ± 0.12	5.14 ^abcd^ ± 0.12	5.14 ^abcd^ ± 0.12	5.14 ^abcd^ ± 0.12	5.14 ^abcd^ ± 0.12	5.14 ^abcd^ ± 0.12	5.14 ^abcd^ ± 0.12	5.14 ^abcd^ ± 0.12	5.14 ^abcd^ ± 0.12	5.14 ^abcd^ ± 0.12	5.14 ^abcd^ ± 0.12	*p* < 0.05
	Day-1	4.99 ^abcde^ ± 0.31	4.98 ^abcde^ ± 0.17	5.01 ^abcde^ ± 0.48	4.71 ^cdeA^ ± 0.33	4.74 ^bcde^ ± 0.15	4.91 ^bcde^ ± 0.15	4.84 ^bcde^ ± 0.31	4.81 ^bcde^ ± 0.19	4.79 ^bcde^ ± 0.08	4.70 ^cde^ ± 0.09	4.39 ^e^ ± 0.04	
	Day-3	5.32 ^abc^ ± 0.07	5.40 ^ab^ ± 0.69	4.91 ^bcde^ ± 0.02	5.07 ^abcd^ ± 0.15	4.98 ^abcde^ ± 0.27	5.11 ^abcd^ ± 0.21	4.99 ^bcde^ ± 0.30	4.88 ^bcde^ ± 0.28	4.99 ^abcde^ ± 0.25	5.01 ^cde^ ± 0.16	4.79 ^bcde^ ± 0.41	
	Day-5	5.26 ^abcdA^ ± 0.02	5.23 ^abcdA^ ± 0.11	5.07 ^abcdA^ ± 0.01	5.21 ^abcdA^ ± 0.20	5.12 ^abcdA^ ± 0.01	5.14 ^abcdA^ ± 0.20	4.93 ^bcdeA^ ± 0.06	4.87 ^bcdeA^ ± 0.07	4.79 ^bcdeA^ ± 0.01	4.85 ^abcde^ ± 0.11	4.61 ^deA^ ± 0.27	
	Day-7	5.59 ^aA^ ± 0.08	5.04 ^abcdeA^ ± 0.03	4.94 ^abcdeA^ ± 0.02	5.01 ^abcdeA^ ± 0.05	4.87 ^bcdeA^ ± 0.06	4.93 ^abcdeA^ ± 0.02	4.81 ^bcdeA^ ± 0.05	4.89 ^bcdeA^ ± 0.01	4.95 ^bcdeA^ ± 0.01	4.89 ^bcde^ ± 0.06	4.82 ^bcdeA^ ± 0.02	
Temp 10 °C	Day-0	5.22 ^ghijk^ ± 0.07	5.22 ^ghijk^ ± 0.07	5.22 ^ghijk^ ± 0.07	5.22 ^ghijk^ ± 0.07	5.22 ^ghijk^ ± 0.07	5.22 ^ghijk^ ± 0.07	5.22 ^ghijk^ ± 0.07	5.22 ^ghijk^ ± 0.07	5.22 ^ghijk^ ± 0.07	5.22 ^ghijk^ ± 0.07	5.22 ^ghijk^ ± 0.07	*p* < 0.05
	Day-1	5.64 ^abcdefghijk^ ± 0.30	5.52 ^cdefghijk^ ± 0.30	5.41 ^efghijk^ ± 0.11	5.72 ^abcdefghijkB^ ± 0.39	5.07 ^ijk^ ± 0.20	4.86 ^k^ ± 0.69	5.07 ^ijk^ ± 0.45	5.23 ^ghijk^ ± 0.21	5.30 ^fghijk^ ± 0.18	5.14 ^hijk^ ± 0.13	4.92 ^jk^ ± 0.28	
	Day-3	6.13 ^abcdefghijk^ ± 0.84	5.91 ^abcdefghijk^ ± 0.59	5.56 ^bcdefghijk^ ± 0.50	5.70 ^abcdefghijk^ ± 0.62	5.59 ^abcdefghijk^ ± 0.59	5.44 ^defghijk^ ± 0.99	5.52 ^cdefghijk^ ± 0.83	5.60 ^abcdefghijk^ ± 0.56	5.70 ^abcdefghijk^ ± 0.56	5.57 ^bcdefghijk^ ± 0.71	5.43 ^defghijk^ ± 0.71	
	Day-5	6.74 ^abcdeB^ ± 0.35	6.47 ^abcdefghiB^ ± 0.29	6.78 ^abcdeB^ ± 0.14	6.83 ^abcdB^ ± 0.10	6.66 ^abcdefB^ ± 0.15	6.68 ^abcdefB^ ± 0.16	6.64 ^abcdefB^ ± 0.05	6.57 ^abcdefgB^ ± 0.24	6.43 ^abcdefghiB^ ± 0.28	6.54 ^abcdefghB^ ± 0.13	6.19 ^abcdefghijkB^ ± 0.17	
	Day-7	6.93 ^abB^ ± 0.32	6.65 ^abcdefB^ ± 0.04	6.89 ^abcB^ ± 0.62	7.00 ^aB^ ± 0.52	6.69 ^abcdefB^ ± 0.07	6.70 ^abcdefB^ ± 0.21	6.45 ^abcdefghiB^ ± 0.13	6.94 ^abB^ ± 0.81	6.78 ^abcdeB^ ± 0.56	6.47 ^abcdefghiB^ ± 0.07	6.28 ^abcdefghijB^ ± 0.02	
Temp 4 °C vs. Temp 10 °C	Day-0	NS	NS	NS	NS	NS	NS	NS	NS	NS	NS	NS	
	Day-1	NS	NS	NS	0.03	NS	NS	NS	NS	0.02	0.01	NS	
	Day-3	NS	NS	NS	NS	NS	NS	NS	NS	NS	NS	NS	
	Day-5	0.03	0.01	0.00	0.00	0.00	0.00	0.00	0.00	0.01	0.00	0.00	
	Day-7	0.01	0.00	0.03	0.02	0.00	0.00	0.00	0.05	0.03	0.00	0.02	

^a–k^ Different letters in each column indicate a significant difference (*p* < 0.05) between the means on the interaction effect of storage with different temperatures at 4 and 10 °C. ^A,B^ Different letters in each column indicate a significant difference (*p* < 0.05) between the means on the same day of storage at 4 and 10 °C. **CNL**: control; CA 0.15: citric acid 0.15%; CA 0.3: citric acid 0.3%; M 0.2: mastic oil 0.2%; M 0.4: mastic oil 0.4%; CH 0.5: chitosan 0.5%; CH 1: chitosan 1%; CACH: citric acid 0.3% + chitosan 1%; CAM: citric acid 0.3% + mastic oil 0.4%; CHM: chitosan 1% + mastic oil 0.4%; CACHM: citric acid 0.3% + chitosan 1% + mastic oil 0.4. Significantly different at (*p* < 0.05); NS: non-significant.

**Table 3:** Effect of antimicrobial agents on the growth of *L. monocytogenes* (log CFU/g) in Guacamole during storage at 4 °C and 10 °C, for 7 days.

	Days	CNL	CA 0.15	CA 0.3	M 0.2	M 0.4	CH 0.5	CH 1	CACH	CAM	CHM	CACHM	*p*-Value
Temp 4 °C	Day-0	4.75 ± 0.79	4.44 ± 0.71	4.44 ± 0.71	4.44 ± 0.71	4.44 ± 0.71	4.44 ± 0.71	4.44 ± 0.71	4.44 ± 0.71	4.44 ± 0.71	4.44 ± 0.71	4.44 ± 0.71	NS
	Day-1	5.37 ± 0.49	5.08 ± 0.51	5.07 ± 0.13	5.05 ± 0.10	4.99 ± 0.10	4.94 ± 0.16	5.00 ± 0.07	5.07 ± 0.16	4.94 ± 0.10	4.95 ± 0.11	4.92 ± 0.20	
	Day-3	5.19 ± 0.32	4.81 ± 0.27	4.84 ± 0.26	4.83 ± 0.34	4.71 ± 0.34	4.73 ± 0.25	4.50 ± 0.40	4.47 ± 0.31	4.53 ± 0.36	4.44 ± 0.43	4.19 ± 0.43	
	Day-5	5.44 ± 0.20	5.21 ± 0.17	5.05 ± 0.13	5.15 ± 0.26	4.96 ± 0.26	5.18 ± 0.02	5.02 ± 0.16	4.91 ± 0.36	5.07 ± 0.12	5.06 ± 0.15	4.79 ± 0.45	
	Day-7	5.43 ^A^ ± 0.35	5.17 ^A^ ± 0.28	5.17 ± 0.18	5.17 ^A^ ± 0.11	5.00 ^A^ ± 0.11	5.09 ± 0.08	4.91 ± 0.13	4.98 ± 0.12	4.97 ± 0.23	5.02 ± 0.11	4.59 ± 0.27	
Temp 10 °C	Day-0	5.41 ± 0.35	5.39 ± 0.25	5.39 ± 0.25	5.39 ± 0.25	5.39 ± 0.25	5.39 ± 0.25	5.39 ± 0.25	5.39 ± 0.25	5.39 ± 0.25	5.39 ± 0.25	5.39 ± 0.25	NS
	Day-1	6.43 ± 0.89	6.35 ± 0.85	6.26 ± 0.79	6.23 ± 0.75	6.34 ± 0.83	6.30 ± 0.81	6.47 ± 1.00	5.95 ± 0.57	6.23 ± 0.75	6.20 ± 0.69	5.94 ± 0.94	
	Day-3	6.20 ± 0.78	5.80 ± 0.94	5.59 ± 1.11	5.59 ± 0.96	5.27 ± 1.06	5.82 ± 0.99	5.55 ± 1.20	5.48 ± 0.99	5.53 ± 0.76	5.35 ± 1.13	5.2 ± 1.07	
	Day-5	5.56 ± 1.07	5.27 ± 1.09	5.03 ± 0.95	5.19 ± 0.80	5.11 ± 0.81	5.06 ± 1.08	5.03 ± 1.01	5.51 ± 0.57	5.08 ± 0.95	5.16 ± 0.94	5.05 ± 0.99	
	Day-7	7.05 ^B^ ± 0.56	6.76 ^B^ ± 0.74	6.40 ± 0.70	6.65 ^B^ ± 0.56	6.57 ^B^ ± 0.67	6.57 ± 0.78	6.54 ± 0.73	5.95 ± 1.56	6.23 ± 0.98	6.58 ± 1.06	6.19 ± 0.78	
Temp 4 °C vs. Temp 10 °C	Day-0	NS	NS	NS	NS	NS	NS	NS	NS	NS	NS	NS	
	Day-1	NS	NS	NS	NS	NS	NS	NS	NS	NS	NS	NS	
	Day-3	NS	NS	NS	NS	NS	NS	NS	NS	NS	NS	NS	
	Day-5	NS	NS	NS	NS	NS	NS	NS	NS	NS	NS	NS	
	Day-7	0.02	0.05	NS	0.03	0.05	NS	NS	NS	NS	NS	NS	

^A,B^ Different letters in each column indicate a significant difference (*p* < 0.05) between the means on the same day of storage at 4 and 10 °C. CNL: control; CA 0.15: citric acid 0.15%; CA 0.3: citric acid 0.3%; M 0.2: mastic oil 0.2%; M 0.4: mastic oil 0.4%; CH 0.5: chitosan 0.5%; CH 1: chitosan 1%; CACH: citric acid 0.3% + chitosan 1%; CAM: citric acid 0.3% + mastic oil 0.4%; CHM: chitosan 1% + mastic oil 0.4%; CACHM: citric acid 0.3% + chitosan 1% + mastic oil 0.4%. Significantly different at (*p* < 0.05); NS: non-significant.

**Table 4:** pH values of Guacamole, treated with different antimicrobials during storage at 4 °C and 10 °C, for 7 days.

	Days	CNL	CA 0.15	CA 0.3	M 0.2	M 0.4	CH 0.5	CH 1	CACH	CAM	CHM	CACHM	*p*-Value
Temp 4 °C	Day-0	7.23 ± 1.75	7.30 ± 1.47	7.32 ± 1.46	7.63 ± 1.60	7.20 ± 1.15	6.85 ± 1.00	6.77 ± 1.18	6.95 ± 1.54	7.03 ± 1.25	6.63 ± 1.05	6.90 ± 1.49	NS
	Day-1	7.57 ± 1.82	7.43 ± 1.65	7.25 ± 1.49	7.36 ± 1.36	7.08 ± 1.03	6.82 ± 0.79	6.44 ± 0.70	6.99 ± 1.37	6.64 ± 0.73	6.20 ± 0.46	5.92 ± 0.24	
	Day-3	7.22 ± 1.54	6.88 ± 1.51	6.51 ± 1.41	7.02 ± 1.07	6.44 ± 0.93	6.53 ± 0.53	6.09 ± 0.50	6.41 ± 1.49	6.26 ± 0.87	5.80 ± 0.38	6.03 ± 0.62	
	Day-5	6.45 ± 1.25	6.11 ± 0.76	5.95 ± 0.75	5.95 ± 0.62	5.94 ± 0.56	5.82 ± 0.64	5.74 ± 0.75	5.78 ± 0.85	5.88 ^A^ ± 0.41	5.51 ± 0.35	5.65 ± 0.69	
	Day-7	7.21 ± 1.89	6.82 ± 1.51	6.61 ± 1.14	6.69 ^A^ ± 1.03	6.24 ± 0.98	6.21 ± 0.91	5.69 ^A^ ± 0.56	6.47 ± 1.11	5.89 ± 0.75	5.35 ^A^ ± 0.28	5.39 ^A^ ± 0.14	
Temp 10 °C	Day-0	6.32 ± 0.21	6.00 ± 0.76	6.11 ± 0.10	6.34 ± 0.24	6.07 ± 0.01	6.35 ± 0.30	6.22 ± 0.12	5.84 ± 0.08	6.03 ± 0.14	5.96 ± 0.14	5.84 ± 0.22	NS
	Day-1	6.16 ± 0.12	5.98 ± 0.31	5.96 ± 0.24	6.06 ± 0.13	5.98 ± 0.11	5.83 ± 0.04	5.85 ± 0.01	5.83 ± 0.10	6.04 ± 0.04	5.70 ± 0.04	5.74 ± 0.19	
	Day-3	5.44 ± 0.43	5.21 ± 0.35	5.25 ± 0.26	5.13 ± 0.22	5.28 ± 0.16	5.46 ± 0.07	5.25 ± 0.25	5.17 ± 0.27	5.17 ± 0.26	5.24 ± 0.26	5.33 ± 0.23	
	Day-5	5.18 ± 0.48	5.07 ± 0.43	5.08 ± 0.31	4.86 ± 0.46	4.88 ± 0.39	4.90 ± 0.30	4.84 ± 0.24	4.88 ± 0.33	4.87 ^B^ ± 0.11	4.98 ± 0.11	4.81 ± 0.19	
	Day-7	4.71 ± 0.62	4.56 ± 0.67	4.51 ± 0.62	4.53 ^B^ ± 0.54	4.53 ± 0.55	4.53 ± 0.32	4.46 ^B^ ± 0.36	4.47 ± 0.55	4.69 ± 0.37	4.41 ^B^ ± 0.37	4.56 ^B^ ± 0.37	
Temp 4 °C vs. Temp 10 °C	Day-0	NS	NS	NS	NS	NS	NS	NS	NS	NS	NS	NS	
	Day-1	NS	NS	NS	NS	NS	NS	NS	NS	NS	NS	NS	
	Day-3	NS	NS	NS	NS	NS	NS	NS	NS	NS	NS	NS	
	Day-5	NS	NS	NS	NS	NS	NS	NS	NS	0.04	NS	NS	
	Day-7	NS	NS	NS	0.05	NS	NS	0.04	NS	NS	0.03	0.05	

^A,B^ Comparison between the microbial populations at 4 °C and 10 °C. CNL: control; CA 0.15: citric acid 0.15%; CA 0.3: citric acid 0.3%; M 0.2: mastic oil 0.2%; M 0.4: mastic oil 0.4%; CH 0.5: chitosan 0.5%; CH 1: chitosan 1%; CACH: citric acid 0.3% + chitosan 1%; CAM: citric acid 0.3% + mastic oil 0.4%; CHM: chitosan 1% + mastic oil 0.4%; CACHM: citric acid 0.3% + chitosan 1% + mastic oil 0.4%. Significantly different at (*p* < 0.05); NS: non-significant.

## Data Availability

The original contributions presented in the study are included in the article, further inquiries can be directed to the corresponding author.
